# Low-normal FT4 in early pregnancy as an independent risk factor for GDM: a large-scale retrospective cohort study

**DOI:** 10.3389/fendo.2026.1762118

**Published:** 2026-04-14

**Authors:** Jin Yuan, Xiaohan Su, Haofan Shi, Yu Meng

**Affiliations:** 1 International Peace Maternity and Child Health Hospital, School of Medicine, Shanghai Jiao Tong University, Shanghai, China; 2 Shanghai Key Laboratory of Embryo Original Diseases, Shanghai, China

**Keywords:** FT4, GDM, parity, pre-pregnancy BMI, TSH

## Abstract

**Objective:**

To investigate the role of early pregnancy thyroid function in gestational diabetes mellitus (GDM) development and its influencing factors.

**Methods:**

This large-scale retrospective cohort study assessed the associations between early pregnancy thyroid hormones and GDM subtypes, as well as their non-linear relationship with oral glucose tolerance test (OGTT) glucose levels, using multivariate logistic regression and restricted cubic spline models. Subgroup analyses were conducted within the normal thyroid function range to evaluate the risk associated with low-normal FT4 levels.

**Results:**

A total of 40,682 pregnant women were included and classified into four groups based on glucose levels: isolated fasting hyperglycemia (IFH), isolated post-load hyperglycemia (IPH), combined hyperglycemia (CH), and normal glucose tolerance (NGT). Free thyroxine (FT4) showed strong capability in differentiating among the subtypes, while thyroid-stimulating hormone (TSH) had limited effects. Multivariate and non-linear analyses showed a J-shaped association between FT4 and fasting/1-hour OGTT glucose, with strong protection below 15.4 pmol/L. In contrast, TSH showed weaker associations without a clear threshold effect. Importantly, low-normal FT4 (11.6–15.4 pmol/L), even within the normal range, independently increased GDM risk, especially in nulliparous and overweight/obese women.

**Conclusion:**

FT4 is an independent risk factor for GDM, with parity and pre-pregnancy BMI serving as important effect modifiers. Even the low-normal FT4 levels are associated with a higher risk of developing GDM and macrosomia.

## Introduction

Gestational diabetes mellitus (GDM) is a common glucose intolerance disorder during pregnancy, classified as an endocrine metabolic disease. Its prevalence is rising, and according to the International Diabetes Federation’s Diabetes Atlas (2025), GDM has been a major global public health issue. GDM increases the risk of maternal complications and adverse outcomes, such as hypertension, preeclampsia, cesarean section, and neonatal respiratory distress syndrome (1, 2). It may also affect the newborn’s heart, indicating a risk of cardiac remodeling (3). Additionally, GDM raises the lifetime risk of type 2 diabetes in mothers (4). At the pathological level, thyroid dysfunction and diabetes share common metabolic pathways and interact through insulin resistance and inflammation (5). As a result, the potential link between thyroid diseases and GDM has gained attention (6, 7). However, the specific role of early pregnancy thyroid function in GDM development remains unclear.

### Subjects

This retrospective cohort study was conducted at a tertiary hospital in Shanghai. Medical records of women with singleton pregnancies who received complete prenatal care and delivered between July 2013 and December 2016 were retrieved. After applying inclusion and exclusion criteria, eligible cases were selected for analysis. The study protocol was approved by the ethics committee, and informed consent was waived due to the use of anonymized data from routine clinical practice. All procedures followed institutional and ethical standards. Participants were eligible for inclusion if they met all of the following criteria: (1) singleton pregnancy; (2) Han Chinese ethnicity; (3) residence in an iodine-sufficient area; (4) thyroid function testing performed during early pregnancy (11–13 weeks of gestation); (5) completion of a 75-gram oral glucose tolerance test (OGTT) at 24–28 weeks of gestation; and (6) delivery at the study hospital. Exclusion criteria were defined as any of the following: (1) pre-existing diabetes or diagnosis of diabetes at the first prenatal visit; (2) history of thyroid disorders, including hyperthyroidism, hypothyroidism, or subclinical hypothyroidism, or the presence of goiter on physical examination; (3) use of medications potentially affecting thyroid function or glucose metabolism before or during pregnancy, such as thyroid hormones, antithyroid drugs, or glucocorticoids; (4) presence of other major systemic diseases that could significantly influence thyroid or glucose metabolism; (5) multiple pregnancy; or (6) non-Chinese nationality.

## Methods

### Data collection

During early pregnancy (11–13 weeks), fasting venous blood samples were collected for serum separation and measurement of thyroid-stimulating hormone (TSH), free thyroxine (FT4), and thyroid peroxidase antibodies (TPOAb) using the Architect i2000 immunoassay analyzer (Abbott, Chicago, USA), following the manufacturer’s protocol. Blood samples were centrifuged within 6 hours of collection. TPOAb ≥ 5.61 IU/mL was considered positive. At mid-pregnancy (24–28 weeks), all participants underwent a standard 75-gram oral glucose tolerance test (OGTT) after at least 8 hours of fasting. Blood samples were collected at baseline, 1 hour, and 2 hours post-glucose intake, and glucose levels were measured using the glucose oxidase method. Maternal baseline information, including age, gravidity, parity, education level, height, and weight, was collected at the first prenatal visit to calculate pre-pregnancy body mass index (BMI). Newborn sex was confirmed from delivery records. The primary endpoint of this study was the occurrence of GDM diagnosed at 24–28 weeks of gestation according to the International Association of Diabetes and Pregnancy Study Groups (IADPSG) criteria (8). Secondary analyses were conducted to explore the associations between FT4 levels and pregnancy outcomes. The study aimed to assess the relationship between early-pregnancy thyroid function and the risk of mid-pregnancy OGTT abnormalities and GDM.

### Definition and reference ranges

Following guidelines from the Endocrine Society and the American Thyroid Association (9), we established pregnancy-specific reference ranges for the first trimester (11–13 weeks) based on a local population. The 2.5th and 97.5th percentiles of TSH and FT4 levels were used to determine the reference range. GDM diagnosis followed the IADPSG criteria (8), with any of the following thresholds: fasting glucose ≥ 5.1 mmol/L, 1-hour glucose ≥ 10.0 mmol/L, or 2-hour glucose ≥ 8.5 mmol/L. BMI categories were: Underweight (BMI < 18.5 kg/m²), Normal weight (18.5 ≤ BMI < 25 kg/m²), Overweight (25 ≤ BMI < 30 kg/m²), and Obesity (BMI ≥ 30 kg/m²) (10–12). We classified GDM into three distinct subtypes based on glucose tolerance test results (13, 14): (1) Impaired Fasting Glucose (IFG), abnormal fasting glucose with normal 1hPG and 2hPG; (2) Impaired Glucose Tolerance (IGT), elevated 1hPG and/or 2hPG with normal fasting glucose; and (3) Combined Hyperglycemia (CH), characterized by abnormalities in both FPG and either 1hPG or 2hPG levels.

### Statistical analysis

Data were analyzed using SPSS version 21.0 (IBM Corp., Armonk, NY) and R software version 3.6.1 (R Development Core Team, 2019; http://www.r-project.org). Continuous variables with normal distribution are presented as mean ± standard deviation and compared using t-tests. Non-normally distributed variables are expressed as median (IQR) and compared using the Mann–Whitney U test. Categorical variables are presented as percentages and compared using the chi-square test. Stratified multivariable logistic regression was used to assess the association between FT4 levels and GDM risk, adjusting for maternal and clinical characteristics, with interaction effects also evaluated. The model was adjusted for maternal age, pre-pregnancy BMI, parity, newborn sex, IVF and TPOAb status. We also assessed potential multicollinearity among covariates using variance inflation factors (VIF). All VIF values were close to 1 (maximum VIF = 1.14), indicating no significant multicollinearity among the variables. To explore potential non-linear associations between thyroid hormones and glucose levels, restricted cubic spline (RCS) models were constructed using the “rms” package in R. The number and placement of knots were determined based on the Akaike Information Criterion (AIC) to achieve optimal model fit. To further examine potential threshold effects, a two-piecewise linear regression (segmented regression) model was applied. The optimal inflection point was determined using a data-driven approach, and model fit between the one-line linear model and the two-piecewise linear model was compared using a likelihood ratio test. Odds ratios (ORs) and 95% confidence intervals (CIs) were calculated. Restricted cubic splines (RCS) and threshold effect analyses assessed the impact of first-trimester FT4 and TSH on glucose levels during mid-pregnancy OGTT. Forest plots were generated using R. Statistical significance was set at a two-sided p-value < 0.05.

## Results

### Population characteristics

**Figure 1:**
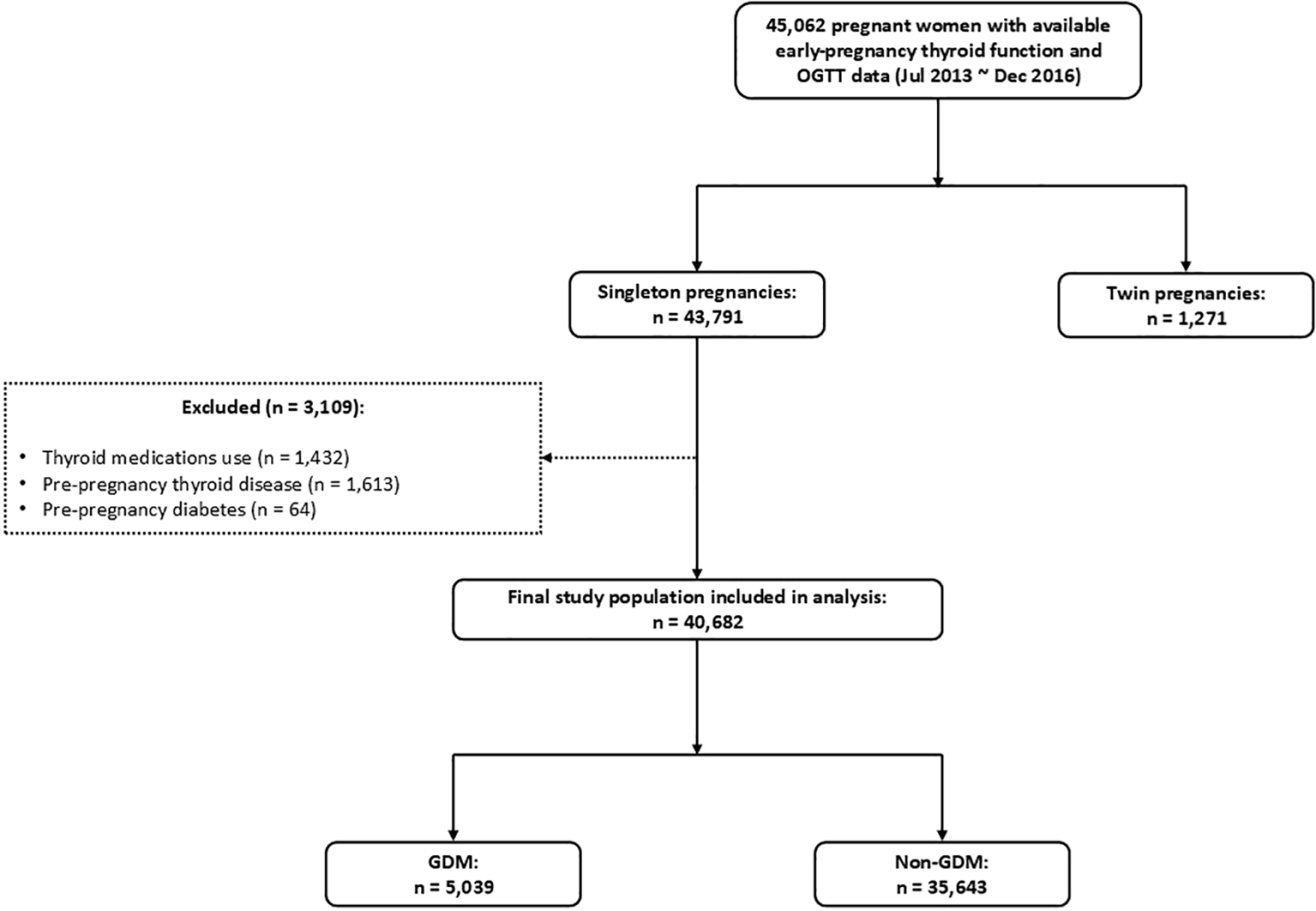
Flowchart of participant selection in the study.

A total of 45,062 pregnant women who delivered at our hospital between July 2013 and December 2016 with available early-pregnancy thyroid function measurements and OGTT data were initially identified from the hospital electronic medical record system. After applying predefined exclusion criteria, a final cohort of 40,682 women was included for analysis. **Figure 1** presents the flowchart of participant selection. After excluding twin pregnancies (n = 1,271), 43,791 singleton pregnancies remained. Among these, 3,109 women were excluded due to thyroid medication use (n = 1,432), pre-pregnancy thyroid disease (n = 1,613), or pre-pregnancy diabetes (n = 64). Consequently, 40,682 euthyroid singleton pregnant women were included in the final analysis. This population was further categorized into women with GDM (n = 5,039) and those without GDM (n = 35,643). Baseline characteristics of the two groups are presented in **Supplementary Table 1**. No statistical comparisons were conducted, as potential differences were accounted for in subsequent multivariable analyses.

### Comparison of FT4 and TSH levels in early pregnancy across GDM subtypes and normal glucose tolerance

A total of 40,682 pregnant women were included in this study, with 5,039 diagnosed with GDM. Based on the characteristics of glucose abnormalities, GDM was divided into three subgroups that may have different pathophysiological mechanisms: isolated fasting hyperglycemia (IFH, n = 398), isolated post-load hyperglycemia (IPH, n = 4,012), and combined hyperglycemia (CH, n = 629). The remaining 35,643 women formed the normal glucose tolerance (NGT) group. Chi-square tests showed significant differences in FT4 and TSH levels between the NGT group and each GDM subgroup (FT4: χ² = 55.27, P < 0.001; TSH: χ² = 16.46, P < 0.001). Median FT4 levels were lower in all GDM subgroups compared to the NGT group, with the CH subgroup showing the most significant reduction. Median TSH levels varied less across groups, although statistically significant differences were still observed.

To explore the associations between FT4, TSH, and GDM risk, multivariate logistic regression was performed (**Table 1**). After adjusting for maternal age, BMI, education level, parity, newborn sex, IVF and TPOAb status, FT4 was found to be a strong protective factor for GDM and its subtypes. Each unit increase in FT4 was associated with a 7% lower risk of IFH (aOR = 0.93, 95% CI: 0.86–0.99, P = 0.037), a 4% lower risk of IPH (aOR = 0.96, 95% CI: 0.94–0.98, P < 0.001), and a 20% lower risk of CH (aOR = 0.80, 95% CI: 0.76–0.85, P < 0.001). These findings suggest that low FT4 levels are closely associated with the development of GDM and its various subtypes, with the strongest association observed for CH. In contrast, TSH showed more limited protection, with significant effects only for IPH (aOR = 0.89, 95% CI: 0.85–0.94, P < 0.001) and CH (aOR = 0.83, 95% CI: 0.74–0.93, P = 0.002), and no significant association with IFH (aOR = 0.86, 95% CI: 0.74–1.00, P = 0.051). This suggests that TSH may mainly play a protective role in GDM subtypes involving post-load glucose abnormalities (IPH and CH). Given that different GDM subtypes may reflect distinct underlying mechanisms of glucose dysregulation, we further examined the value of FT4 and TSH in differentiating between these subtypes. The results showed FT4 was a key marker for distinguishing CH from IFH and IPH, with significantly lower FT4 levels in CH (IFH vs. CH: aOR = 1.12, 95% CI: 1.04–1.22, P = 0.005; IPH vs. CH: aOR = 1.18, 95% CI: 1.11–1.26, P < 0.001). However, FT4 did not differ significantly between IFH and IPH (aOR = 0.97, P = 0.403). In comparison, TSH showed no significant differences across any of the GDM subtype comparisons, indicating that it may lack the ability to differentiate between specific GDM phenotypes. In summary, low FT4 levels are consistently associated with an increased risk of all GDM subtypes, particularly with the CH subtype, which involves both fasting and post-load glucose abnormalities and may represent a more severe metabolic disturbance. Compared with TSH, FT4 appears to have stronger predictive and discriminatory value in early pregnancy, likely due to its stability and direct reflection of thyroid hormone status.

**Table 1:** Multivariate analysis of early pregnancy FT4 and TSH levels across GDM subtypes and normal glucose tolerance.

**Reference group**	**Comparison group**	**Variable**	**β**	**S.E.**	**Z**	**P-value**	**aOR (95% CI)**
NGT	IFH	FT4	-0.08	0.04	-2.09	0.037*	0.93 (0.86–0.99)
		TSH	-0.15	0.08	-1.95	0.051	0.86 (0.74–1.00)
	IPH	FT4	-0.04	0.01	-4.02	<0.001*	0.96 (0.94–0.98)
		TSH	-0.11	0.02	-4.71	<0.001*	0.89 (0.85–0.94)
	CH	FT4	-0.22	0.03	-7.62	<0.001*	0.80 (0.76–0.85)
		TSH	-0.18	0.06	-3.15	0.002*	0.83 (0.74–0.93)
IPH	IFH	FT4	-0.03	0.04	-0.84	0.403	0.97 (0.90–1.04)
		TSH	-0.05	0.08	-0.57	0.567	0.96 (0.82–1.12)
CH	IFH	FT4	0.12	0.04	2.82	0.005*	1.12 (1.04–1.22)
		TSH	0.06	0.09	0.59	0.554	1.06 (0.88–1.27)
	IPH	FT4	0.17	0.03	5.49	<0.001*	1.18 (1.11–1.26)
		TSH	0.07	0.06	1.14	0.254	1.07 (0.95–1.21)

Multivariate logistic regression models adjusted for maternal age, BMI, education, parity, IVF, newborn sex, and TPOAb. β: regression coefficient; S.E.: standard error; Z: Z statistic; P: p-value; aOR (95% CI): adjusted odds ratio with 95% confidence interval. Statistical significance defined as P < 0.05.

### Nonlinear relationship and threshold effects of early thyroid function on OGTT glucose levels

**Figure 2:**
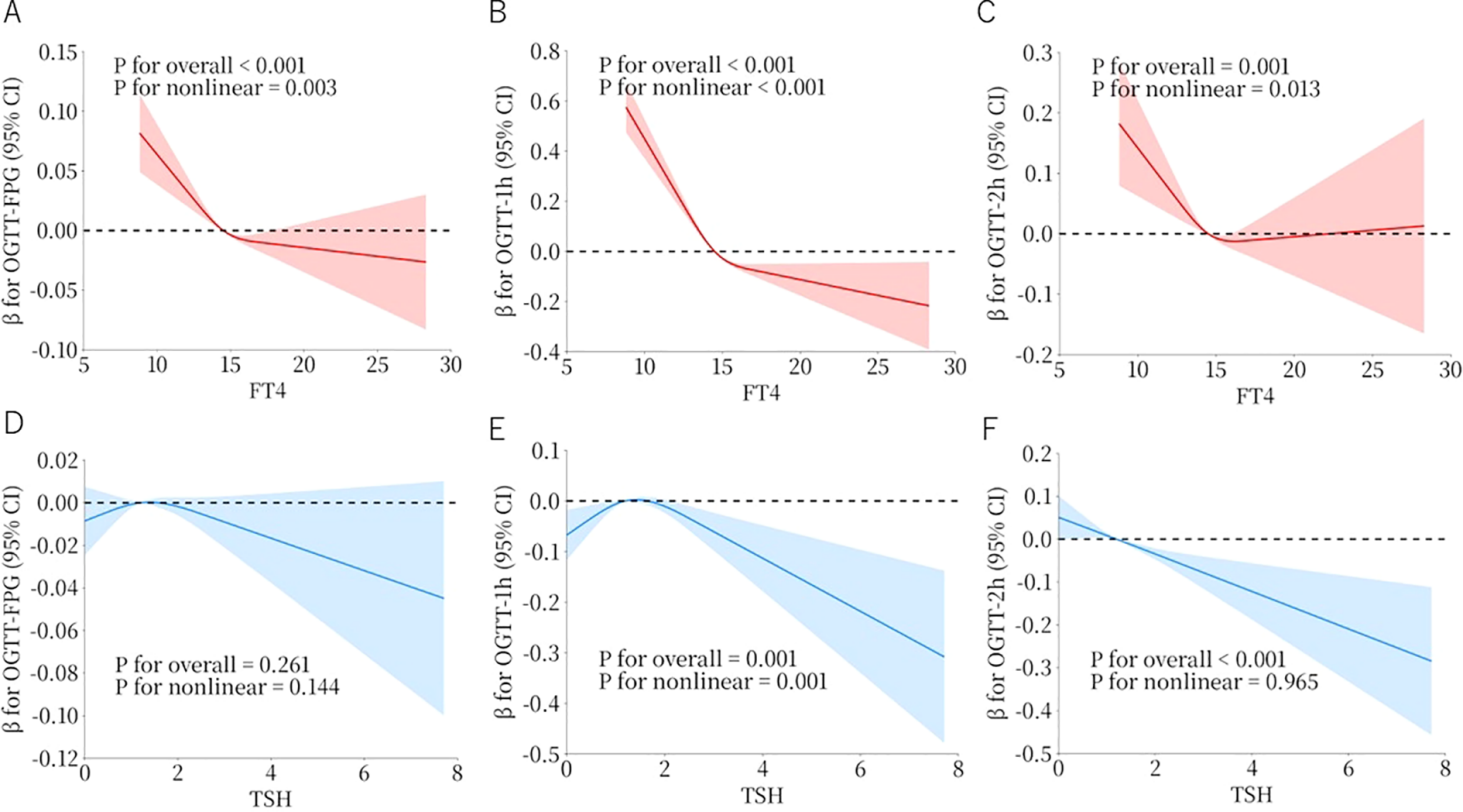
Restricted cubic spline (RCS) models showing the nonlinear associations between FT4 and TSH levels and OGTT-derived glucose values. The Y-axis represents the regression coefficient (β) and its 95% confidence interval. The X-axis shows thyroid function indicators (FT4 or TSH). **(A–C)** show the associations of FT4 with fasting, 1-hour, and 2-hour glucose levels, respectively; **(D–F)** show the corresponding associations for TSH. The RCS models were adjusted for the following variables: maternal age, BMI, education, parity, IVF, newborn sex, and TPOAb.

Based on multivariate analysis, FT4 and TSH were identified as independent protective factors against GDM. However, the log-linear assumptions of logistic regression may not fully capture the complex, non-linear relationship between thyroid hormones and glucose metabolism. Therefore, we used RCS (as shown in **Figure 2**), along with threshold effect analyses (as shown in **Table 2**) to examine associations between first trimester thyroid function parameters (FT4 and TSH) and mid pregnancy OGTT glucose levels (fasting, 1 hour, and 2 hour). We found that FT4 and TSH show distinct association patterns with glucose measures. FT4 exhibited a significant J-shaped association with fasting glucose and 1-hour OGTT glucose (P for nonlinear = 0.003 and <0.001, respectively), as well as a threshold effect (P for likelihood ratio test < 0.001 and 0.016, respectively), with the threshold identified at 15.4 pmol/L. Below this threshold, FT4 was inversely associated with glucose risk (fasting: OR = 0.81, 95% CI 0.76–0.87, P < 0.001; 1 hour: OR = 0.92, 95% CI 0.88–0.96, P < 0.001), but above it, no significant effect was found. For 2-hour glucose, FT4 showed a negative nonlinear trend (p for nonlinear = 0.013), but no threshold effect (P for likelihood ratio test = 0.386). In contrast to FT4, which demonstrated strong associations with clear threshold effects, TSH exhibited a weak and heterogeneous pattern of associations with glucose parameters. Although RCS analysis revealed a nonlinear relationship with 1-hour OGTT glucose (overall P < 0.001), the curve was relatively smooth, with no detectable threshold effect (P for likelihood ratio test = 0.231). By contrast, TSH was linearly and inversely associated with 2-hour glucose (OR = 0.92, P < 0.001), and no significant association was observed with fasting glucose. This limited pattern of associations, along with the absence of threshold effects, further underscores the more central and stage-specific role of FT4 in the regulation of glucose metabolism during pregnancy.

**Table 2:** Threshold effect analysis of first-trimester FT4 and TSH on mid-pregnancy OGTT glucose level.

Glucose	Thyroid function	Standard linear regression model		Two-piecewise linear regression model					
		OR (95%CI)	P	Inflection point	< Inflection point		≥ Inflection point		P for likelihood ratio test
					OR (95%CI)	P	OR (95%CI)	P	
Fasting Blood	FT4	0.90 (0.86 – 0.94)	<0.001*	15.4	0.81 (0.76 – 0.87)	**<0.001**	1.08 (0.99 – 1.19)	0.080	<0.001*
	TSH	0.92 (0.84 – 1.00)	0.063	1.7	0.95 (0.77 - 1.17)	0.636	0.89 (0.74 - 1.07)	0.213	0.846
1-hour post-OGTT	FT4	0.96 (0.94 – 0.98)	0.001*	15.4	0.92 (0.88 – 0.96)	**<0.001**	1.01 (0.96 – 1.07)	0.588	0.016*
	TSH	0.95 (0.91 – 1.00)	0.044*	1.0	1.11 (0.86 - 1.44)	0.424	0.91 (0.85 - 0.98)	**0.014**	0.231
2-hour post-OGTT	FT4	0.97 (0.95 – 0.99)	0.012*	12.7	0.90 (0.79 – 1.03)	0.119	0.98 (0.95 – 1.01)	0.143	0.386
	TSH	0.92 (0.87 – 0.97)	<0.001*	3.1	0.91 (0.85 – 0.97)	**0.003**	0.75 (0.56 – 1.00)	0.050	0.957

Model 1: Standard linear regression model assessing the overall linear association between first-trimester FT4 or TSH and mid-pregnancy OGTT glucose groups. Results are presented as odds ratios (OR) with 95% confidence intervals (CI). Model 2: Two-piecewise linear regression model accounting for potential threshold effects, estimating associations separately below and above the inflection point. Inflection point: The identified cutoff value in Model 2 that separates two different effect intervals of the predictor variable. P for likelihood test: Tests the presence of a significant threshold effect in Model 2; P-value < 0.05 indicates a statistically significant nonlinear segmented relationship. Bold values indicate statistical significance (P < 0.05).

### Low-normal FT4 based on threshold analysis and its association with GDM and pregnancy outcomes

The normal ranges for thyroid function were defined based on the 2.5th–97.5th percentiles. The calculated normal range for FT4 was 11.6–19.5 pmol/L, and for TSH it was 0.03–3.63 mIU/mL. Based on the threshold effect analysis of the association between FT4 and OGTT glucose levels, an inflection point of 15.4 pmol/L was identified. Using this cut-off value, participants with normal thyroid function were further stratified into two groups: a low-normal FT4 group (11.6 ≤ FT4 < 15.4 pmol/L, n = 24,196) and an optimal FT4 group (15.4 ≤ FT4 < 19.5 pmol/L, n = 13,128). Multivariable logistic regression analysis, adjusted for maternal age, parity, pre-pregnancy BMI, education level, newborn sex, IVF conception, and TPOAb status, demonstrated that low-normal FT4 remained an independent risk factor for GDM (OR = 1.13, 95% CI: 1.04–1.23; P = 0.005). As a sensitivity analysis, FT4 levels were further categorized into quartiles. Consistent with the primary threshold-based approach, participants in the lowest FT4 quartile (Q1) exhibited a significantly higher risk of GDM compared with those in the highest quartile (Q4) after adjusting for potential confounders. A significant dose-response trend was observed across quartiles (P for trend < 0.001), further supporting the robustness of the association. Detailed results are presented in **Table 3**.

**Table 3:** Association between maternal early-pregnancy FT4 levels and GDM risk: quartile-based and threshold-based analyses.

Grouping	FT4 group	FT4 range (pmol/L)	Incidence of GDM (%)	Univariate analysis		Multivariate analysis	
				OR (95%CI)	P	aOR (95%CI)	P
Quartiles	**Q4**	15.9 ≤ FT4 ≤ 19.5	10.7	1.00 (Reference)		1.00 (Reference)	
	**Q3**	14.7 ≤ FT4 < 15.9	11.4	1.07 (0.98–1.18)	0.134	1.00 (0.88–1.12)	0.950
	**Q2**	13.7 ≤ FT4 < 14.7	13.1	1.26 (1.15–1.38)	**<0.001**	1.13 (1.01–1.27)	**0.037**
	**Q1**	11.6 ≤ FT4 < 13.7	14.1	1.37 (1.26–1.50)	**<0.001**	1.13 (1.01–1.27)	**0.032**
Threshold	**Optimal FT4 group**	15.4 ≤ FT4 < 19.5	11.0	1.00 (Reference)		1.00 (Reference)	
	**Low-normal FT4 group**	11.6 ≤ FT4 < 15.4	13.1	1.23 (1.15–1.31)	**<0.001**	1.13 (1.04–1.23)	**0.005**

FT4 groups were defined based on the distribution in the study population and analyzed using both quartile-based and threshold-based approaches. Multivariable regression models are adjusted for maternal age, pre-pregnancy BMI, education level, IVF status, newborn sex, TPOAb status, and parity. P for trend across FT4 quartiles was calculated using the Cochran–Armitage test for trend; p < 0.001 (data not shown in table). GDM, gestational diabetes mellitus; FT4, free thyroxine; OR, odds ratio; aOR, adjusted odds ratio; CI, confidence interval. Bold values indicate statistical significance (P < 0.05).

To further evaluate the clinical significance of low-normal FT4, we compared the incidence of adverse pregnancy outcomes between these two groups. After multivariable adjustment, low-normal FT4 was associated with a significantly increased risk of macrosomia (aOR = 1.16, 95% CI: 1.03–1.30, P = 0.014). In contrast, no significant associations were observed for preeclampsia, low birth weight (LBW), or preterm birth. This pattern may be partly explained by the closer relationship between macrosomia and GDM, while all participants were within the euthyroid reference range, potentially attenuating the effect of FT4 on other outcomes less directly related to glucose metabolism. Detailed results, including crude and adjusted analyses for all outcomes, are presented in **Supplementary Table 2**.

### Stratified analyses by maternal and clinical characteristics

To further explore potential effect modifiers, we performed stratified analyses and interaction tests across various maternal and clinical characteristics. The results are presented in **Table 4**. Stratified analyses further revealed that this risk was significant in nulliparous women (OR = 1.17, 95% CI: 1.07–1.29, interaction P = 0.031) and overweight or obese women with a pre-pregnancy BMI ≥ 25 kg/m² (OR = 1.52, 95% CI: 1.16–2.00, interaction P = 0.003). These findings suggest thyroid function screening in high-risk populations may help with the early prevention of GDM.

**Table 4:** Association between FT4 levels and the risk of GDM stratified by maternal and clinical characteristics.

Stratified variable	Proportion (%)	The incidence of GDM (%)		aOR (95%CI)	P	P for interaction
		Low-normal FT4 group	Optimal FT4 group			
All patients	100.0	13.1	11.0	1.13 (1.04 – 1.23)	**0.005**	
Parity						**0.031**
Nulliparas	81.3	12.9	10.6	1.17 (1.07 – 1.29)	**0.001**	
Multiparas	18.7	13.9	13.3	0.95 (0.79 – 1.15)	0.608	
Newborn Sex						0.418
Male	52.4	13.4	10.9	1.17 (1.03 – 1.31)	**0.012**	
Female	47.6	12.8	11.0	1.09 (0.96 – 1.23)	0.169	
IVF						0.772
Without	96.8	12.8	10.7	1.12 (1.03 – 1.23)	**0.010**	
With	3.2	23.1	19.2	1.28 (0.88 – 1.86)	0.205	
TPOAb						0.621
Negative	90.3	13.1	10.9	1.14 (1.04 – 1.24)	**0.005**	
Positive	9.7	13.4	11.7	1.05 (0.79 – 1.39)	0.729	
Age						0.940
< 35y	86.9	11.8	10.1	1.11 (1.02 – 1.22)	**0.023**	
≥ 35y	13.1	20.5	19.1	1.16 (0.94 – 1.44)	0.155	
Prepregnancy BMI						**0.029**
< 25 kg/m²	92.1	11.0	9.8	1.09 (0.99 – 1.19)	0.065	
≥ 25 kg/m²	7.9	26.1	18.6	1.52 (1.16 – 2.00)	**0.003**	

The OR values represent the odds ratio for the Low-normal FT4 group compared to the Optimal FT4 group, which is used as the reference group (OR = 1). Multivariable regression models are adjusted for maternal age, pre-pregnancy BMI, education level, IVF status, newborn sex, TPOAb status, and parity. Percentages are calculated within each subgroup. P values indicate the statistical significance of the association between FT4 group and the risk of GDM within each subgroup. P for interaction assesses whether the association between FT4 group and GDM risk significantly differs across levels of the stratified variable, indicating potential effect modification. aOR: Adjusted Odds Ratio; CI: Confidence Interval. Bold values indicate statistical significance (P < 0.05).

## Discussion

Recent studies have increasingly highlighted the relationship between thyroid function and the risk of gestational diabetes. A recent individual participant data meta-analysis by Osinga et al. (15) reported that, compared with euthyroid pregnant women, isolated hypothyroxinaemia was associated with a higher risk of GDM. Our findings are consistent with this evidence, further supporting a close relationship between lower FT4 levels and the risk of GDM. Importantly, our results extend these observations by demonstrating that even FT4 levels within the normal range, particularly those below 15.4 pmol/L, were associated with an increased risk of GDM. Similar observations were reported in the study by Song et al. (16), which suggested that thyroid function, even within the normal range, is closely related to glucose and lipid metabolism during early pregnancy. Although that study emphasized the FT4/TSH ratio and identified it as an independent risk factor for GDM among women carrying male fetuses, our findings likewise indicate that subtle variations in thyroid function within the euthyroid range may influence metabolic adaptations during pregnancy. Moreover, we identified clinically relevant subgroups (such as nulliparous women and those who were overweight or obese) in whom this association appeared to be stronger. In contrast to FT4, which showed strong associations with glucose parameters and clear threshold effects, TSH exhibited weaker and more heterogeneous associations and showed no significant differences across GDM subtypes. This limited pattern may be partly explained by physiological suppression of TSH by hCG in early pregnancy.

This study suggests FT4 may be a general risk factor for GDM, contributing to both fasting and post-load glucose abnormalities, likely through its roles in regulating hepatic gluconeogenesis, muscle glucose uptake, and lipid metabolism, all of which influence insulin resistance (13, 17, 18). Particularly, the lowest FT4 levels were observed in the CH subgroup. This suggests more profound metabolic dysregulation in this subtype, reflecting β-cell decompensation in the context of combined insulin resistance. It is noteworthy that FT4 levels did not differ significantly between the IFH and IPH subgroups, both of which are characterized by isolated glucose abnormalities. This implies that low FT4 may concurrently impair hepatic insulin sensitivity (19) and peripheral tissue glucose disposal (20), thereby raising the risk for both fasting and postprandial hyperglycemia. Thus, when comparing only IFH and IPH, FT4 as a common upstream risk factor showed similar reductions in both groups, resulting in no significant intergroup difference. In contrast, the association between TSH and GDM was weaker, primarily observed in the IPH and CH subgroups, with no significant discriminative value among GDM subtypes. This suggests that, compared to FT4’s direct metabolic regulatory role, TSH levels mainly reflect the intensity of efforts to maintain thyroid hormone homeostasis, serving as an adaptive signal to metabolic challenges during pregnancy. Therefore, FT4 is likely a more sensitive and reliable marker of thyroid function for GDM risk assessment than TSH. In summary, this study highlights the complex role of thyroid hormones, especially FT4, in GDM development and subtyping. These findings offer a foundation for future research into GDM phenotypic differentiation and precise risk prediction. Additionally, our study reveals a stable, J-shaped nonlinear relationship between first-trimester FT4 levels and mid-pregnancy glucose levels, with a threshold around 15.4 pmol/L. Below this level, FT4 strongly associates with elevated fasting and 1-hour glucose, establishing low-normal FT4 as an independent risk factor for dysglycemia and providing a risk threshold for clinical assessment.

This study reveals a dual regulatory pattern, both marker-specific and time-specific, between early pregnancy thyroid hormones and mid-pregnancy glucose metabolism. FT4 showed a saturation-like relationship with fasting and 1-hour post-load glucose, with its effect being most pronounced within a certain range. Below the threshold of 15.4 pmol/L, FT4 was strongly negatively associated with glucose, indicating its importance in maintaining metabolic function and insulin sensitivity. However, once FT4 levels exceed this threshold, the association disappears, possibly reflecting a plateau in its effect. This J-shaped pattern and threshold together reinforce the central role of thyroid hormones in energy balance regulation. FT4 levels below 15.4 pmol/L may signal early metabolic imbalance (21). For fasting glucose, low FT4 may reflect reduced suppression of hepatic gluconeogenesis, potentially contributing to increased hepatic glucose output (13). For 1-hour glucose, which primarily reflects early postprandial insulin sensitivity and glucose disposal in peripheral tissues, especially skeletal muscle, it is particularly sensitive to changes in FT4. The observed association may be explained by the known effects of inadequate FT4 levels on insulin signaling pathways and GLUT4 transporter function, which can result in insulin resistance and elevated glucose peaks (14, 22). In this study, the same FT4 threshold for both fasting and 1-hour glucose may represent a true biological cutoff. Before this threshold is reached, the body may maintain stable blood glucose levels through compensatory mechanisms, such as increased insulin secretion (23). Once FT4 drops below this level, the concurrent changes in glucose levels could reflect the simultaneous disruption of multiple processes necessary for glucose stability. These processes include the expression of genes like PEPCK and GLUT4 (22), ATP production in cells (24), and insulin signaling in the liver and muscles (25). The simultaneous changes observed in our study may collectively signal the shift from a compensated to a decompensated state. This highlights the critical role of FT4 in maintaining glucose stability during pregnancy. Additionally, the study found that although FT4 was negatively associated with 2-hour plasma glucose, it did not show a clear threshold effect like fasting and 1-hour glucose. This difference may stem from the distinct physiological implications of 2-hour plasma glucose: it not only reflects insulin resistance but also serves as a “stress test” of pancreatic β-cell compensatory insulin secretion following a glucose load (26). Therefore, the complex interaction between the metabolically unfavorable background set by low FT4 levels and the individual’s β-cell functional reserve may smooth out or obscure any sharp inflection point, resulting in a negative association without a distinct threshold. This pattern may reflect β-cell decompensation in some individuals, though direct assessment of β-cell function was not performed in this study. Compared to FT4, the associations between TSH and glycemic indicators were weaker and inconsistent, further supporting the idea that FT4 is more physiologically relevant than TSH in the direct regulation of peripheral metabolism (27). The persistent linear inverse correlation observed between TSH and 2-hour postprandial glucose may reflect the intrinsic link between the set point of the HPT axis and the body’s long-term baseline glucose clearance efficiency. As a pituitary hormone, TSH primarily reflects the regulatory status of the HPT axis, rather than the direct biological effects of thyroid hormones on peripheral tissues (22). Its influence on glucose metabolism is relatively weak under basal metabolic conditions, but the correlation with dynamic glucose levels, such as 2-hour post-load glucose, strengthens under metabolic stress like glucose loading. This suggests that TSH levels may indirectly represent the impact of thyroid function on insulin secretion adaptability (28), highlighting the potential role of thyroid regulation during metabolic challenges (29). In summary, FT4, as the direct physiological effector, exhibits a saturable kinetic pattern indicating its direct action, while TSH, as an indirect marker of the HPT axis status, may reflect the long-term, systemic influence of the HPT axis on the metabolic environment. Together, these findings suggest a complex picture of how the thyroid system may finely tune glucose metabolism during pregnancy.

Most previous studies on thyroid function and GDM have focused on overt abnormalities. Zou et al. reported that maternal thyroid dysfunction in the context of GDM was associated with an increased risk of adverse pregnancy outcomes, including preterm birth, hypertensive disorders, and fetal overgrowth, highlighting the need for combined screening of thyroid function and glucose metabolism (30). Our study extends these observations to women with euthyroid thyroid function but low-normal FT4 levels, showing that low-normal FT4 was associated with an increased risk of macrosomia. In a fundamental shift of perspective, this study systematically demonstrates that low-normal FT4 levels, even within the euthyroid range, independently increase GDM risk, challenging the traditional assumption of “normal equals safe” and highlighting the greater public health importance of monitoring the normal range. While overt thyroid dysfunction affects only a small percentage (around 5%) of the pregnant population, the low-normal FT4 subgroup comprises about 60% of our study participants. This means more than half of clinically euthyroid pregnant women may be at elevated risk for GDM. Although the increase in relative risk for an individual is modest, the overall impact cannot be neglected due to the substantial number of pregnant women affected. Solely focusing on the minority with overt dysfunction would overlook this vast, vulnerable population. Therefore, a strategic shift in focus from identifying “abnormality” to “optimization within the normal range” represents a necessary and critical breakthrough for the effective population-wide prevention of GDM. More importantly, this association is not uniform across all groups. Our stratified analysis further revealed that the link between low-normal FT4 levels and GDM risk is particularly pronounced among nulliparas women and those who were overweight or obese before pregnancy. These findings underscore the potential for refining GDM risk stratification and developing targeted monitoring strategies for these susceptible subgroups.

Pre-pregnancy BMI is a well-established, modifiable risk factor for GDM across diverse populations (31, 32). Its mechanism involves impaired lipolysis in GDM, which elevates hepatic glucose output and exacerbates insulin resistance, a pathway confirmed in animal studies (33). Our moderation analysis further identified BMI as a critical effect modifier: for the first time, we observed that the GDM risk linked to low FT4 levels was significantly heightened in individuals with BMI >25 kg/m². While prior research emphasizes the protective role of FT4 in GDM and the delicate regulation of glucose metabolism by thyroid homeostasis (34), and suggests reduced thyroid hormone sensitivity may be an adaptive response to energy surplus in GDM (35), the crosstalk between adipose tissue and muscle is also important. Adipokines from adipose tissue modulate insulin sensitivity (36), while myokines from skeletal muscle influence adipose function (37). Our findings integrate these pathways, proposing that obesity may amplify the disruptive effect of low FT4 on this adipose–muscle axis, underscoring the deep interrelationship among pre-pregnancy BMI, thyroid hormones, and metabolic phenotype. We also observed a stronger association between low-normal FT4 and GDM risk in nulliparous than multiparous women, suggesting their metabolic systems are more sensitive to low FT4 due to lack of prior pregnancy adaptation. This aligns with Ingram et al., who found nulliparous women’s insulin sensitivity is more vulnerable to visceral fat accumulation, while multiparous women exhibit better metabolic adaptability (38). We propose a “double-hit” framework: the untrained metabolic system of nulliparous women is simultaneously challenged by low FT4 and pregnancy-induced insulin resistance, easily exceeding their compensatory capacity and increasing GDM risk. Multiparous women, with prior metabolic adaptation, show greater resilience. This aligns with domestic research (39) indicating a lower GDM risk in multiparous women compared to nulliparous women and with De Groot et al., who reported a higher GDM prevalence among primiparas, especially under metabolic stressors like age and obesity (9). Future prevention strategies for pregnancy-related metabolic disorders should fully account for the fundamental metabolic differences between nulliparous and multiparous women, as well as among women with normal versus elevated pre-pregnancy BMI, to implement more targeted and precise risk management.

### Strengths and limitations

This study offers several key strengths. Firstly, it introduces a new perspective by highlighting that even low-normal FT4 levels in pregnant women with normal thyroid function are independently associated with a higher risk of GDM. Notably, this risk is particularly higher in nulliparous and overweight or obese women, suggesting a previously unrecognized high-risk subgroup. This finding supports a shift from diagnosing overt dysfunction to identifying and managing subtle physiological risk states. Secondly, the results indicate that FT4 may be a more sensitive and clinically relevant marker than TSH in predicting GDM. As an active hormone involved in peripheral metabolism FT4 fluctuations may more directly affect insulin sensitivity and glucose regulation, while TSH may be less responsive due to its regulatory role and feedback mechanisms. Finally, the assessment of first-trimester thyroid function as a prospective measure prior to the diagnosis of mid-pregnancy GDM ensures the temporal sequence of causality. Furthermore, extensive multivariable adjustment was applied, which minimized confounding bias and confirmed a robust independent association. This study also has several limitations. Firstly, we only explored the association between thyroid function in early pregnancy and the risk of GDM. Due to the retrospective nature of the study, longitudinal measurements of thyroid function across pregnancy (e.g., during mid and late pregnancy) were not available, which limited our ability to assess the dynamic relationship between thyroid function and glucose metabolism throughout pregnancy. Secondly, as a retrospective study, residual confounding cannot be ruled out, as important factors such as diet, physical activity, hCG levels, gestational age at thyroid testing, and detailed family history were unavailable for adjustment. Additionally, individual urinary iodine data were lacking. While Shanghai is not considered iodine-deficient overall (40), regional and individual variations in iodine status may influence the observed association between FT4 and GDM (41), potentially confounding the results. It is also important to note that although multiple covariates were included in the regression models, the large sample size and substantial number of GDM events help mitigate the risk of model overfitting. However, as with any observational analysis, some degree of overfitting cannot be entirely ruled out. Further studies are needed in larger multicenter cohorts to validate these findings and to include longitudinal measurements of thyroid function, hCG levels, iodine status, and glucose metabolism throughout pregnancy. Furthermore, exploring early lifestyle interventions or thyroid function monitoring in high-risk groups, such as nulliparous or obese women with low FT4, could enhance targeted prevention strategies for GDM.

## Data Availability

The data analyzed in this study is subject to the following licenses/restrictions: The data that support the findings of this study are available on request from the corresponding author. The data are not publicly available due to privacy or ethical restrictions. Requests to access these datasets should be directed to Mengyu, mengyu_1224@126.com.

## References

[1] Modzelewski R Stefanowicz-Rutkowska MM Matuszewski W Bandurska-Stankiewicz EM . Gestational diabetes mellitus-recent literature review. J Clin Med. (2022) 11:5736. doi: 10.3390/jcm11195736 36233604 PMC9572242

[2] Karkia R Giacchino T Shah S Gough A Ramadan G Akolekar R . Gestational diabetes mellitus: Association with maternal and neonatal complications. Med Kaunas Lith. (2023) 59:2096. doi: 10.3390/medicina59122096 38138200 PMC10744613

[3] Di Bernardo SC Lava SAG Epure AM Younes SE Chiolero A Sekarski N . Consequences of gestational diabetes mellitus on neonatal cardiovascular health: MySweetHeart Cohort study. Pediatr Res. (2023) 94:231–8. doi: 10.1038/s41390-022-02390-4 36443400 PMC10356590

[4] Diaz-Santana MV O’Brien KM Park YMM Sandler DP Weinberg CR . Persistence of risk for type 2 diabetes after gestational diabetes mellitus. Diabetes Care. (2022) 45:864–70. doi: 10.2337/dc21-1430 35104325 PMC9016728

[5] Tilici DM Paun DL Arnautu AM Mirica A Duta C Costea M . The intricate relationship between thyroid disorders and type 2 diabetes—a narrative review. Diabetology. (2025) 6:41. doi: 10.3390/diabetology6050041 41725453

[6] Biondi B Kahaly GJ Robertson RP . Thyroid dysfunction and diabetes mellitus: Two closely associated disorders. Endocr Rev. (2019) 40:789–824. doi: 10.1210/er.2018-00163 30649221 PMC6507635

[7] Chauhan A Patel SS . Thyroid hormone and diabetes mellitus interplay: Making management of comorbid disorders complicated. Horm Metab Res Horm Stoffwechselforschung Horm Metab. (2024) 56:845–58. doi: 10.1055/a-2374-8756 39159661

[8] Metzger BE Gabbe SG Persson B Buchanan TA Catalano PA International Association of Diabetes and Pregnancy Study Groups Consensus Panel . International association of diabetes and pregnancy study groups recommendations on the diagnosis and classification of hyperglycemia in pregnancy. Diabetes Care. (2010) 33:676–82. doi: 10.2337/dc09-1848 20190296 PMC2827530

[9] De Groot L Abalovich M Alexander EK Amino N Barbour L Cobin RH . Management of thyroid dysfunction during pregnancy and postpartum: an Endocrine Society clinical practice guideline. J Clin Endocrinol Metab. (2012) 97:2543–65. doi: 10.1210/jc.2011-2803 22869843

[10] Haam JH Kim BT Kim EM Kwon H Kang JH Park JH . Diagnosis of obesity: 2022 update of clinical practice guidelines for obesity by the Korean Society for the Study of Obesity. J Obes Metab Syndr. (2023) 32:121–9. doi: 10.7570/jomes23031 37386771 PMC10327686

[11] Jensen MD Ryan DH Apovian CM Ard JD Comuzzie AG Donato KA . 2013 AHA/ACC/TOS guideline for the management of overweight and obesity in adults: a report of the American College of Cardiology/American Heart Association Task Force on Practice Guidelines and The Obesity Society. J Am Coll Cardiol. (2014) 63:2985–3023. doi: 10.1016/j.jacc.2013.11.004 24239920

[12] Garvey WT Mechanick JI Brett EM Garber AJ Hurley DL Jastreboff AM . American Association of Clinical Endocrinologists and American College of Endocrinology comprehensive clinical practice guidelines for medical care of patients with obesity. Endocr Pract Off J Am Coll Endocrinol Am Assoc Clin Endocrinol. (2016) 22:1–203. doi: 10.4158/EP161365.GL 27219496

[13] Kotzaeridi G Blätter J Eppel D Rosicky I Linder T Geissler F . Characteristics of gestational diabetes subtypes classified by oral glucose tolerance test values. Eur J Clin Invest. (2021) 51:e13628. doi: 10.1111/eci.13628 34120352 PMC8459269

[14] Athanasiadou KI Paschou SA Markozannes G Vasileiou V Kanouta F Mitropoulou M . Gestational diabetes mellitus subtypes according to oral glucose tolerance test and pregnancy outcomes. Endocrine. (2025) 90:95–103. doi: 10.1007/s12020-025-04329-1 40569563 PMC12464073

[15] Osinga JAJ Derakhshan A Karachaliou M Poppe KG Warringa L Verdonk K . Association of gestational thyroid function and thyroid autoimmunity with gestational diabetes: a systematic review and individual participant meta-analysis. Lancet Diabetes Endocrinol. (2025) 13:651–61. doi: 10.1016/S2213-8587(25)00068-3 40609565

[16] Song S Zhang Y Qiao X Duo Y Xu J Zhang J . Thyroid FT4-to-TSH ratio in the first trimester is associated with gestational diabetes mellitus in women carrying male fetus: a prospective bi-center cohort study. Front Endocrinol. (2024) 15:1427925. doi: 10.3389/fendo.2024.1427925 39678197 PMC11637856

[17] Garduño-Garcia JJ Camarillo Romero E Loe Ochoa A Romero-Figueroa S Huitron Bravo G Torres García R . Thyroid function is associated with insulin resistance markers in healthy adolescents with risk factors to develop diabetes. Diabetol Metab Syndr. (2015) 7:16. doi: 10.1186/s13098-015-0011-x 25780389 PMC4361132

[18] Teixeira SS Panveloski-Costa AC Carvalho A Monteiro Schiavon FP Ruiz Marque AC Campello RS . Thyroid hormone treatment decreases hepatic glucose production and renal reabsorption of glucose in alloxan-induced diabetic Wistar rats. Physiol Rep. (2016) 4:e12961. doi: 10.14814/phy2.12961 27655796 PMC5037915

[19] Liu CH Zeng QM Hu TY Huang Y Song Y Guan H . Resmetirom and thyroid hormone receptor-targeted treatment for metabolic dysfunction-associated steatotic liver disease (MASLD). Portal Hypertens Cirrhosis. (2025) 4:66–78. doi: 10.1002/poh2.100 40777891 PMC12327538

[20] Navale AM Paranjape AN . Glucose transporters: physiological and pathological roles. Biophys Rev. (2016) 8:5–9. doi: 10.1007/s12551-015-0186-2 28510148 PMC5425736

[21] Cicatiello AG Di Girolamo D Dentice M . Metabolic effects of the intracellular regulation of thyroid hormone: Old players, new concepts. Front Endocrinol. (2018) 9:474. doi: 10.3389/fendo.2018.00474 30254607 PMC6141630

[22] Mullur R Liu YY Brent GA . Thyroid hormone regulation of metabolism. Physiol Rev. (2014) 94:355–82. doi: 10.1152/physrev.00030.2013 24692351 PMC4044302

[23] Subramanian V Sherman AS Holst JJ Knop FK Vilsbøll T Bagger JI . Evaluating the role of alpha cell dysregulation in the progression to type 2 diabetes using mathematical simulations. Diabetologia. (2025) 68(11):2595–608. doi: 10.1007/s00125-025-06524-1 40924110 PMC12534288

[24] Vaitkus JA Farrar JS Celi FS . Thyroid hormone mediated modulation of energy expenditure. Int J Mol Sci. (2015) 16:16158–75. doi: 10.3390/ijms160716158 26193258 PMC4519944

[25] Moeller LC Cao X Dumitrescu AM Seo H Refetoff S . Thyroid hormone mediated changes in gene expression can be initiated by cytosolic action of the thyroid hormone receptor β through the phosphatidylinositol 3-kinase pathway. Nucl Recept Signal. (2006) 4:nrs.04020. doi: 10.1621/nrs.04020 16862226 PMC1513074

[26] Mengozzi A Tricò D Nesti L Petrie J Højlund K Mitrakou A . Disruption of fasting and post-load glucose homeostasis are largely independent and sustained by distinct and early major beta-cell function defects: a cross-sectional and longitudinal analysis of the relationship between insulin sensitivity and cardiovascular risk (RISC) study cohort. Metabolism. (2020) 105:154185. doi: 10.1016/j.metabol.2020.154185 32061908

[27] Fitzgerald SP Bean NG Hennessey JV Falhammar H . Thyroid testing paradigm switch from thyrotropin to thyroid hormones—future directions and opportunities in clinical medicine and research. Endocrine. (2021) 74:285–9. doi: 10.1007/s12020-021-02851-6 34449031 PMC8497305

[28] Baeyens L Hindi S Sorenson RL German MS . β‐cell adaptation in pregnancy. Diabetes Obes Metab. (2016) 18:63–70. doi: 10.1111/dom.12716 27615133 PMC5384851

[29] Gavrila A Hollenberg AN . The hypothalamic-pituitary-thyroid axis: Physiological regulation and clinical implications. In: Luster M Duntas LH Wartofsky L , editors.The thyroid and its diseases: A comprehensive guide for the clinician. Springer International Publishing, Cham (2019). p. 13–23. doi: 10.1007/978-3-319-72102-6_2, PMID:

[30] Zou C Shen Q Yang Y Liao Y Du Q . Association of maternal thyroid function and gestational diabetes with pregnancy outcomes: a retrospective cohort study. Front Endocrinol. (2025) 16:1555409. doi: 10.3389/fendo.2025.1555409 40547523 PMC12178882

[31] Zhang S Liu H Li N Dong W Li W Wang L . Relationship between gestational body mass index change and the risk of gestational diabetes mellitus: a community-based retrospective study of 41,845 pregnant women. BMC Pregnancy Childbirth. (2022) 22:336. doi: 10.1186/s12884-022-04672-5 35440068 PMC9020000

[32] Mnatzaganian G Woodward M McIntyre HD Ma L Yuen N He F . Trends in percentages of gestational diabetes mellitus attributable to overweight, obesity, and morbid obesity in regional Victoria: an eight-year population-based panel study. BMC Pregnancy Childbirth. (2022) 22:95. doi: 10.1186/s12884-022-04420-9 35105311 PMC8809044

[33] Mittelman SD Bergman RN . Inhibition of lipolysis causes suppression of endogenous glucose production independent of changes in insulin. Am J Physiol Endocrinol Metab. (2000) 279:E630–7. doi: 10.1152/ajpendo.2000.279.3.E630 10950832

[34] Chen GD Gou XY Pang TT Li PS Zhou ZX Lin DX . Associations between thyroid function and gestational diabetes mellitus in Chinese pregnant women: a retrospective cohort study. BMC Endocr Disord. (2022) 22:44. doi: 10.1186/s12902-022-00959-y 35189861 PMC8862524

[35] Liu Z Li G Wu Y Zhang D Zhang S Hao YT . Increased central and peripheral thyroid resistance indices during the first half of gestation were associated with lowered risk of gestational diabetes—analyses based on Huizhou Birth Cohort in South China. Front Endocrinol. (2022) 13:806256. doi: 10.3389/fendo.2022.806256 35345468 PMC8957094

[36] Rabe K Lehrke M Parhofer KG Broedl UC . Adipokines and insulin resistance. Mol Med Camb Mass. (2008) 14:741–51. doi: 10.2119/2008-00058.Rabe 19009016 PMC2582855

[37] Havekes B Sauerwein HP . Adipocyte-myocyte crosstalk in skeletal muscle insulin resistance; is there a role for thyroid hormone? Curr Opin Clin Nutr Metab Care. (2010) 13:641–6. doi: 10.1097/MCO.0b013e32833e341d 20689414

[38] Ingram KH Hunter GR James JF Gower BA . Central fat accretion and insulin sensitivity: differential relationships in parous and nulliparous women. Int J Obes. (2017) 41:1214–7. doi: 10.1038/ijo.2017.104 28465610 PMC5555115

[39] Dai Y Wang Y Chen M Lin Q Situ J Yu Y . Impact of parity on gestational diabetes mellitus in Chinese women: a retrospective cohort study. BMC Pregnancy Childbirth. (2025) 25:520. doi: 10.1186/s12884-025-07620-1 40307716 PMC12042609

[40] Tian W Yan W Liu Y Zhou F Wang H Sun W . The status and knowledge of iodine among pregnant women in Shanghai. Biol Trace Elem Res. (2021) 199:4489–97. doi: 10.1007/s12011-021-02587-4 33462796

[41] Yu Z Zheng C Zheng W Wan Z Bu Y Zhang G . Mild-to-moderate iodine deficiency in a sample of pregnant women and salt iodine concentration from Zhejiang province, China. Environ Geochem Health. (2020) 42:3811–8. doi: 10.1007/s10653-020-00640-0. PMID: 32596780

