# Supplementary material for: Low-normal FT4 in early pregnancy as an independent risk factor for GDM: a large-scale retrospective cohort study
Source: Front Endocrinol (Lausanne). 2026 Apr 14;17:1762118. doi: 10.3389/fendo.2026.1762118 (PMC13122590; doi:10.3389/fendo.2026.1762118)
Supplement: Supplementary file 1 [file Table1.docx]

**Table S1. Baseline Characteristics of the Study Subjects by GDM Status**

| Variables | | Total  (n = 40,682) | Non-GDM  (n = 35,643) | GDM  (n = 5,039) |
| --- | --- | --- | --- | --- |
| Age, M (Q₁, Q₃) | | 30.0 (28.0, 32.0) | 30.0 (27.0, 32.0) | 31.0 (29.0, 34.0) |
| Pre-pregnancy BMI, M (Q₁, Q₃) | | 20.6 (19.1, 22.5) | 20.5 (19.1, 22.3) | 21.6 (19.9, 23.7) |
| Parity, n (%) | |  |  |  |
|  | Nulliparas | 33,013 (81.2%) | 29,040 (81.5%) | 3,973 (78.9%) |
|  | Multiparas | 7,669 (18.8%) | 6,603 (18.5%) | 1,066 (21.1%) |
| Newborn Sex, n (%) | |  |  |  |
|  | Male | 21,178 (52.1%) | 18,524 (52.0%) | 2,654 (52.7%) |
|  | Female | 19,504 (47.9%) | 17,119 (48.0%) | 2,385 (47.3%) |
| IVF, n (%) | |  |  |  |
|  | Without IVF | 39,353 (96.7%) | 34,608 (97.1%) | 4,745 (94.2%) |
|  | With IVF | 1,329 (3.3%) | 1,035 (2.9%) | 294 (5.8%) |
| TPOAb Status, n (%) | |  |  |  |
|  | Negative | 36,566 (89.9%) | 32,061 (90.0%) | 4,505 (89.4%) |
|  | Positive | 4,116 (10.1%) | 3,582 (10.0%) | 534 (10.6%) |
| Maternal Education Level, n (%) | |  |  |  |
|  | Below bachelor’s degree | 9,260 (22.8%) | 7,957 (22.3%) | 1,303 (25.9%) |
|  | Bachelor’s degree or higher | 31,422 (77.2%) | 27,686 (77.7%) | 3,736 (74.1%) |

*Values are presented as median (Q1, Q3) for continuous variables and number (percentage) for categorical variables. BMI: body mass index; IVF: in vitro fertilization; TPOAb: thyroid peroxidase antibody; GDM: gestational diabetes mellitus. No statistical comparisons were made, as differences were adjusted for in multivariable models.*
