# Supplementary material for: Low-normal FT4 in early pregnancy as an independent risk factor for GDM: a large-scale retrospective cohort study
Source: Front Endocrinol (Lausanne). 2026 Apr 14;17:1762118. doi: 10.3389/fendo.2026.1762118 (PMC13122590; doi:10.3389/fendo.2026.1762118)
Supplement: Supplementary file 2 [file Table2.docx]

**Table S2. Associations of low-normal FT4 with adverse pregnancy outcomes**

| Outcome | Incidence n (%) | Univariate Analysis | | | | | Multivariate Analysis | | | | |
| --- | --- | --- | --- | --- | --- | --- | --- | --- | --- | --- | --- |
|  |  | **β** | **SE** | **Z** | **P** | **OR (95% CI)** | **β** | **SE** | **Z** | **P** | **aOR (95% CI)** |
| Preeclampsia | 556 (2.3%) | 0.16 | 0.08 | 2.05 | **0.040** | 1.17 (1.01–1.36) | 0.01 | 0.09 | 0.10 | 0.920 | 1.01 (0.84–1.21) |
| Macrosomia | 1564 (6.46%) | 0.21 | 0.05 | 4.49 | **<0.001** | 1.23 (1.13–1.35) | 0.15 | 0.06 | 2.45 | **0.014** | 1.16 (1.03–1.30) |
| LBW | 646 (2.67%) | 0.01 | 0.07 | 0.21 | 0.833 | 1.01 (0.89–1.16) | -0.03 | 0.08 | -0.41 | 0.680 | 0.97 (0.82–1.13) |
| Preterm birth | 1225 (5.06%) | 0.08 | 0.05 | 1.61 | 0.107 | 1.09 (0.98–1.20) | 0.07 | 0.06 | 1.08 | 0.282 | 1.07 (0.95–1.21) |

*Reference group (Optimal FT4) was defined as 15.4 ≤ FT4 < 19.5 pmol/L. Low-normal FT4 group was defined as 11.6 ≤ FT4 < 15.4 pmol/L, based on the threshold derived from GDM risk analysis. Incidence n (%) represents the number and proportion of participants in the low-normal FT4 group who experienced the respective outcome. β, SE, Z, and P represent regression coefficient, standard error, Z-statistic, and P-value from univariate or multivariate logistic regression. OR = crude odds ratio; aOR = adjusted odds ratio (95% confidence intervals). Multivariable regression models are adjusted for maternal age, pre-pregnancy BMI, education level, IVF status, newborn sex, TPOAb status, and parity. LBW = Low Birth Weight, defined as birth weight <2500 g.*
